# Yellow Fever Outbreak — Kongo Central Province, Democratic Republic of the Congo, August 2016

**DOI:** 10.15585/mmwr.mm6612a5

**Published:** 2017-03-31

**Authors:** John O. Otshudiema, Nestor G. Ndakala, Elande-taty K. Mawanda, Gaston P. Tshapenda, Jacques M. Kimfuta, Loupy-Régence N. Nsibu, Abdou S. Gueye, Jacob Dee, Rossanne M. Philen, Coralie Giese, Christopher S. Murrill, Ray R. Arthur, Benoit I. Kebela

**Affiliations:** ^1^Epidemic Intelligence Service Program, CDC; ^2^Meningitis and Vaccine Preventable Diseases Branch, Division of Bacterial Diseases, National Center for Immunization and Respiratory Diseases, CDC; ^3^Field Epidemiology and Laboratory Training Program, Kinshasa, Democratic Republic of the Congo; ^4^Division Provinciale de la Santé, Kongo Central Province, Ministry of Health, Democratic Republic of the Congo; ^5^Direction de Lutte contre la Maladie, Ministry of Health, Kinshasa, Democratic Republic of the Congo; ^6^Zone de Santé de Nsona-Mpangu, Kongo Central Province, Democratic Republic of the Congo; ^7^Division of Global Health Protection, Center for Global Health, CDC; ^8^Division of Global HIV and TB, Center for Global Health, CDC; ^9^Global Immunization Division, Center for Global Health, CDC.

On April 23, 2016, the Democratic Republic of the Congo’s (DRC’s) Ministry of Health declared a yellow fever outbreak. As of May 24, 2016, approximately 90% of suspected yellow fever cases (n = 459) and deaths (45) were reported in a single province, Kongo Central Province, that borders Angola, where a large yellow fever outbreak had begun in December 2015. Two yellow fever mass vaccination campaigns were conducted in Kongo Central Province during May 25–June 7, 2016 and August 17–28, 2016. In June 2016, the DRC Ministry of Health requested assistance from CDC to control the outbreak. As of August 18, 2016, a total of 410 suspected yellow fever cases and 42 deaths were reported in Kongo Central Province. Thirty seven of the 393 specimens tested in the laboratory were confirmed as positive for yellow fever virus (local outbreak threshold is one laboratory-confirmed case of yellow fever). Although not well-documented for this outbreak, malaria, viral hepatitis, and typhoid fever are common differential diagnoses among suspected yellow fever cases in this region. Other possible diagnoses include Zika, West Nile, or dengue viruses; however, no laboratory-confirmed cases of these viruses were reported. Thirty five of the 37 cases of yellow fever were imported from Angola. Two-thirds of confirmed cases occurred in persons who crossed the DRC-Angola border at one market city on the DRC side, where ≤40,000 travelers cross the border each week on market day. Strategies to improve coordination between health surveillance and cross-border trade activities at land borders and to enhance laboratory and case-based surveillance and health border screening capacity are needed to prevent and control future yellow fever outbreaks.

Yellow fever is an arthropod-borne flavivirus, transmitted in urban outbreaks primarily by *Aedes aegypti* mosquitoes. Signs and symptoms take 3–6 days to develop and include fever, chills, headache, and muscle aches. In general, worldwide approximately 15% of persons with yellow fever develop serious illness that can lead to bleeding, shock, organ failure, and death (*1*). The historic case fatality rate for yellow fever in Africa is approximately 20% (*2*).

Yellow fever vaccine is safe and effective (*2*). Approximately 1.5 million doses were administered in two mass vaccination campaigns conducted in Kongo Central Province; these campaigns were estimated to have reached 99% administrative vaccination coverage (the number of vaccine doses administered divided by the most recent census estimates for the targeted population). The CDC team visited Kongo Central Province during August and September 2016, reviewed yellow fever surveillance data reported in the DRC Integrated Disease Surveillance and Response system, assessed health facilities and border ports of entry, interviewed health and border surveillance officers, and made recommendations for prevention and control.

A suspected yellow fever case was defined by the DRC Ministry of Health (adapted from the World Health Organization’s standard case definition) as acute onset of fever, followed by jaundice within 14 days of symptom onset. Laboratory-confirmed cases were defined as 1) detection in serum of yellow fever virus–specific immunoglobulin M and yellow fever–specific neutralizing antibodies or yellow fever virus nucleic acid by polymerase chain reaction, or 2) isolation of yellow fever virus from a blood specimen. In response to the outbreak, the DRC Ministry of Health implemented yellow fever case–based surveillance with immediate notification and field investigation requirements, including collection of blood specimens, ascertainment of vaccination status, and documentation of travel history. On the basis of travel history and location of exposure, laboratory-confirmed cases with no previous vaccination history were classified as imported (from another country) or autochthonous. Health facilities reported all suspected yellow fever cases to the health zone office in their jurisdiction; health zone reports were compiled by the Kongo Central Province Health Division. All blood specimens were sent from affected health zones to the National Institute of Biomedical Research in Kinshasa, DRC’s capital, which serves as the national reference laboratory. Surveillance and laboratory data were tabulated in Epi Info and descriptive analyses were performed using statistical software.

From January 4 to August 18, 2016, a total of 410 suspected yellow fever cases, including 42 (10.2%) deaths were reported in Kongo Central Province. Blood specimens from 393 (98.5%) suspected cases were collected and tested for yellow fever virus; 37 (9.4%) were positive, 346 (88.0%) were negative (n = 325) or discarded because of recent vaccination (21), and results for 10 (2.5%) were inconclusive (Table). Among the 37 confirmed cases, 32 (86.5%) were serologically confirmed and five (13.5%) were confirmed by detection of yellow fever virus nucleic acid by polymerase chain reaction or isolation of yellow fever virus.

**TABLE:** Classification of reported yellow fever cases, by confirmation status, outcome, and location of exposure — Kongo Central Province, Democratic Republic of the Congo, January 4–August 18, 2016

Indicator	No. (%)
Reported suspected cases	410 (100)
Deaths* (CFR)	42/410 (10.2)
Specimens collected and tested during investigation*	393/410 (98.5)
Negative and discarded cases^†^	346/393 (88.0)
Unclassified/inconclusive cases^†^	10/393 (2.5)
Laboratory-confirmed cases^†^	37/393 (9.4)
Imported cases^§^	35/37 (94.6)
Autochthonous cases^§^	2/37 (5.4)
**Deaths (CFR)^§^**	**8/37 (21.6)**

**Abbreviation:** CFR = case fatality ratio.

* Among suspected cases.

^†^ Among suspected cases with submitted specimen.

^§^ Among laboratory-confirmed cases.

The median age of persons with laboratory-confirmed cases of yellow fever was 31 years (range = 0–72 years) and 86.4% were male; eight deaths occurred among confirmed cases (case-fatality ratio = 21.6%) (Table). Thirty five (94.5%) laboratory-confirmed yellow fever cases occurred in persons who had been in Angola in the 14 days preceding illness onset and were thus classified as imported from Angola; the other two (5.4%) were classified as autochthonous. The highest numbers of laboratory-confirmed cases were reported in March and April 2016 (Figure 1) and began to decline before the vaccination campaigns. No additional cases were confirmed after June 27, 2016.

**FIGURE 1:**
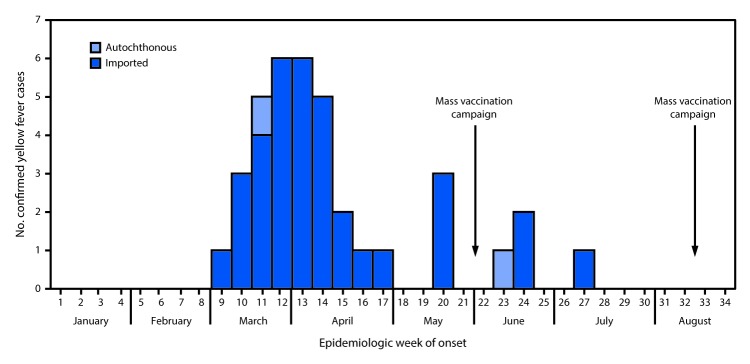
Confirmed yellow fever cases, by week of onset and importation status — Kongo Central Province, Democratic Republic of the Congo, January 3–August 18, 2016 (N = 37)

Within Kongo Central Province, laboratory-confirmed cases were reported in eight health zones, seven (87.5%) of which border Angola. The highest incidence of laboratory-confirmed yellow fever cases in Kongo Central Province was in Nsona-Mpangu Health Zone (13 cases per 100,000 population; 15 laboratory-confirmed cases) (Figure 2). The market city of Lufu in the Nsona-Mpangu Health Zone accounted for 23 of 35 (65.7%) laboratory-confirmed cases imported from Angola. Lufu is situated on the DRC-Angola border and ≤40,000 travelers cross the border every week on market day (in a 10-hour period ≥65 persons per minute are seen crossing). At the time of the outbreak, four health professionals were assigned to identify travelers with unexplained fever and jaundice consistent with the suspected yellow fever case definition, obtain travel histories, and check yellow fever vaccination certificates.

**FIGURE 2:**
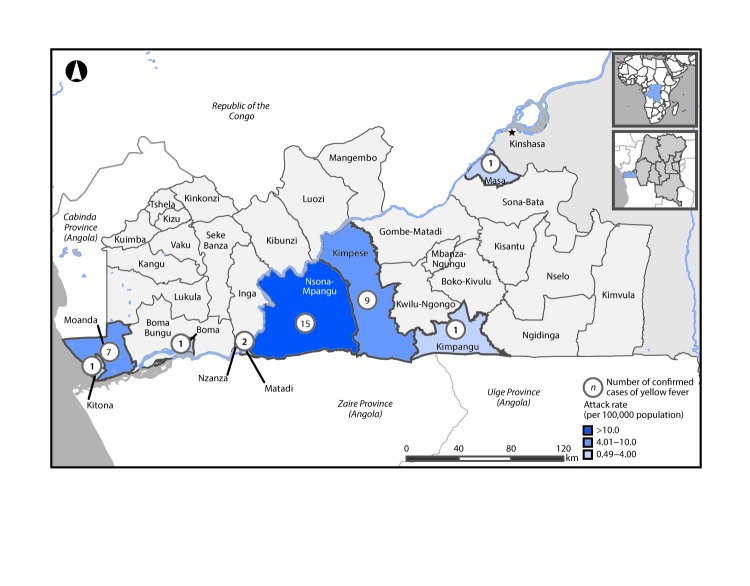
Number of confirmed yellow fever cases, by health zone — Kongo Central Province, Democratic Republic of the Congo, January 3–August 18, 2016 (N = 37)

In some remote areas of Kongo Central Province, because of the absence of correct supplies and standard operating procedures for specimen collection, inappropriate and nonsterile 5-mL vacuum tubes were used to collect blood. The average time between blood collection at health facilities in Kongo Central Province and receipt of specimens at the reference laboratory was 4 days (range = 1–7 days).

## Discussion

The yellow fever outbreak in Kongo Central Province was associated with high population mobility across a porous border and was characterized by wide geographic spread in health zones bordering Angola. The Angola-DRC border market city of Lufu was the main port of entry for persons with laboratory-confirmed cases imported from Angola and accounted for two-thirds of imported confirmed cases in the province. Resources allocated for control and screening of ≤40,000 travelers through Lufu each day were insufficient; similar border issues were described during the 2014 Ebola virus outbreak in West Africa (*3*).

Nsona-Mpangu Health Zone recorded the highest yellow fever incidence rate in Kongo Central Province (13 cases per 100,000 population) and in the DRC overall. In contrast, incidence rates in the two neighboring Angolan border provinces of Zaire and Uige were estimated to be substantially lower than those in Nsona-Mpangu (approximately 0.21–2.99 per 100,000) (*4*). In addition, a higher case fatality rate among persons with laboratory-confirmed yellow fever cases was reported in Kongo Central Province (21.6%) compared with the case fatality rate reported by the World Health Organization for Angola (13.6% [121 cases per 884 persons]) (*5*). Although the outbreak was controlled through enhanced surveillance and mass vaccination campaigns, with no laboratory-confirmed cases reported since July 27, the risk for yellow fever transmission persists because of increases in transmission during the annual rainy season and intense cross-border trade activities.

The findings in this report are subject to at least two limitations, both of which were surveillance challenges in Kongo Central Province highlighted by field visits. First, there were insufficient human resources to conduct adequate case-based surveillance and health screening in a context of substantial population movement across porous borders. Second, laboratory supplies for blood specimen collection were lacking and the system for transporting blood specimens from health facilities in Kongo Central Province to the reference laboratory in Kinshasa (about 300 miles) was inefficient. Blood specimens should be sent in a cooler or ordinary domestic vacuum flask to the reference laboratory as soon as possible and not later than 24 hours after collection. Delays in transportation, inadequate supplies for collection of specimens, and inappropriate handling of specimens might have compromised the quality of some specimens, possibly resulting in a low case confirmation rate (9.4%; the confirmation rate during the yellow fever outbreak in Angola was approximately 27%).

To successfully prevent and control future yellow fever outbreaks, laboratory- and case-based surveillance needs to be strengthened, cross-border coordination improved, and vaccination coverage increased. In addition, more complete yellow fever vector data are needed to better characterize the prevalence and epidemiology of yellow fever outbreaks in this forested border region.

Yellow fever is preventable through vaccination. The DRC Ministry of Health requires all persons aged ≥9 months crossing the border to show proof of yellow fever vaccination upon arrival or to be vaccinated, but the high population mobility and ineffective screening capacity at Kongo Central Province’s remote land ports of entry overwhelmed the screening system. Reinforced coordination between health surveillance and cross-border trade activities at land crossing borders is needed to prevent yellow fever transmission.

**Summary**

**What is already known about this topic?**

Border areas with high population mobility and intense trade activities can foster outbreaks such as yellow fever, particularly in settings where vaccination coverage and health screening capacity are not optimal. In December 2015, a large yellow fever outbreak began in Angola, bordering the Democratic Republic of the Congo (DRC).

**What is added by this report?**

In February 2016, a yellow fever outbreak was declared in DRC; approximately 90% of suspected cases and deaths occurred in Kongo Central Province. Thirty seven of the 393 specimens tested received laboratory confirmation of yellow fever virus; 35 of these 37 cases were imported from neighboring Angola. Most imported cases occurred in persons who crossed the DRC-Angola border at a single market city, where ≤40,000 travelers cross the border each week on market day, overwhelming the border health screening program. Insufficient laboratory supplies and delayed transport of specimens to the laboratory compromised case confirmation.

**What are the implications for public health practice?**

Reinforcement of coordination between enhanced laboratory and case-based surveillance, with health border screening and cross-border trade activities, is necessary at land crossing borders to prevent and control future yellow fever outbreaks.
